# Clinical evaluation of photochromic nanoparticle tattoo ink: safety, tolerability, and performance of rewritable intradermal implants

**DOI:** 10.1186/s12951-026-04755-x

**Published:** 2026-07-02

**Authors:** Keith McCurdy, Jesse L. Butterfield, Hyejin Kwon, Emily Gardner, Madison Jordan, Matthew Ganser, Michael J. Mouncer, Jon Osis, Christophe Clement, Adarsh V. Mudgil, Carson J. Bruns

**Affiliations:** 1 HYPRSKN Inc., Boulder, 80309 CO USA; 2 ATLAS Institute, University of Colorado Boulder, Boulder, 80309 CO USA; 3 Rady Department of Mechanical Engineering, University of Colorado Boulder, Boulder, 80309 CO USA; 4 Mudgil Dermatology, New York, 10011 NY USA

**Keywords:** Photochromic, Nanoparticles, Tattoo, Ink, Intradermal, Implants, Smart materials, Clinical evaluation, Biocompatibility, Dermatology

## Abstract

‘Smart tattoos’ based on stimulus-responsive nanoparticles have emerged as a promising platform for intradermal biosensing and dynamic optical interfaces, yet translation to safe and sustained use in human skin remains largely untested. We report a pilot clinical evaluation of Magic Ink, a rewritable photochromic tattoo ink comprising nano-engineered pigments that switch reversibly between nearly colorless and magenta states under visible and ultraviolet light, enabling permanent intradermal tattoos to be visually erased and restored on demand. Preclinical studies established third-party-verified sterility and non-cytotoxicity, confirmed nanoparticle size/morphology and shear-thinning rheological behavior suitable for intradermal delivery, and demonstrated robust photochromic performance with rapid switching kinetics and high fatigue resistance. In a paired, within-subject study, 11 participants received matched Magic Ink and commercial ‘Standard Ink’ control tattoos. Healing outcomes were assessed by clinical inspection (days 7 & 21), participant surveys through day 21, and longitudinal photographic documentation over five months. No adverse events were observed (0/11 per group). Dermatologist assessments and participant-reported symptoms of pain, redness, and itch did not differ significantly between Magic Ink and Standard Ink tattoos. Magic Ink tattoos remained nearly imperceptible in the deactivated state yet became readily visible upon UV activation after at least one year *in vivo*, demonstrating durable retention of photoswitchable function in human skin. These results indicate that photo-rewritable tattoo nanomaterials can function as tolerable intradermal implants over the timescales examined, and support their further evaluation for dynamic or ‘disappearing’ tattoos in medical (e.g., radiotherapy treatment localization and biopsy site marking in dermatology), cosmetic and paramedical (e.g., permanent makeup and micropigmentation), and body art applications.

**Graphical abstract:**

**Figure:**
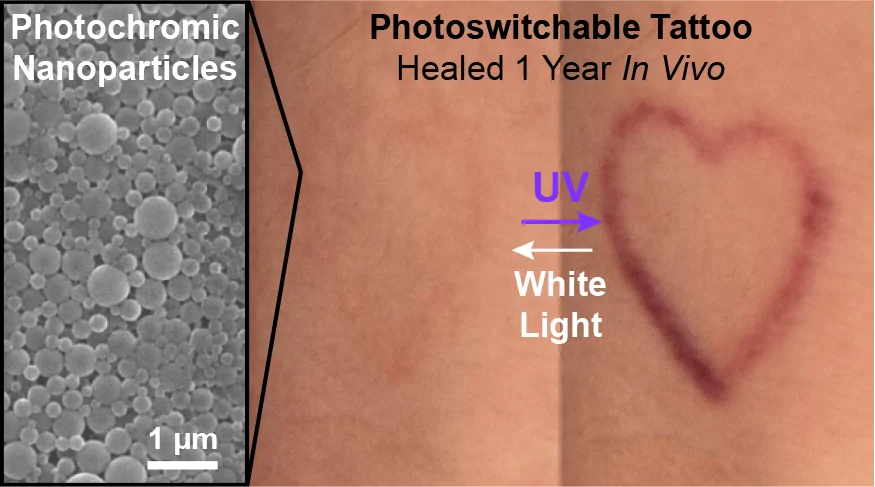

## Introduction

Tattoos are permanent skin markings that represent a large multibillion-dollar industry in which hundreds of millions of people participate around the world. Body art tattoos are considered pro-social [1], with potential benefits that include improved self-esteem [2, 3] and self-identity [4], communicative value [5], and emotional support for patients who have undergone surgery [6, 7] or psychological trauma. [8–10] While most tattoos are obtained for cosmetic body art or permanent makeup, various medical tattoos are also administered routinely, for example in biopsy site identification in dermatology [11–13] and treatment localization in radiation therapy [14, 15]. Medical aesthetics tattoos [16, 17] are also commonly used to camouflage pigment disorders [18–20], scars [17, 21–24], hair loss [25, 26], or tissue reconstruction [17, 27, 28]. These treatments can have high customer satisfaction rates (>90%) [17] and improve patient quality of life [29].

Tattooing is a process that deposits pigment granules in the dermis by any method that allows them to bypass the epidermal barrier of the skin, such as puncturing, scratching or cutting. As evidenced by its ubiquitous worldwide practice for millennia [30], tattooing is a reasonably safe, affordable, and tolerable process, but not without risks. Aesthetic risks include blurring [31], fading [32, 33], “blowout” [34, 35], and color change [36, 37], as well as non-allergic reactions that lead to thickened or raised regions of tattooed skin [35, 38]. Health risks include allergic reactions (mostly occurring in red tattoos), papulo-nodular reactions, bacterial infections, photosensitivity, pain syndrome, and lymphopathy [39, 40]. Although many tattoo pigments are known [41, 42] to contain or degrade into hazardous compounds, most tattoos do not yield health complications. An investigation [43] of 234 dermatological patients with tattoos found that only 5 (2.1%) suffered adverse complications, none of which were life threatening. Despite much investigation there is no evidence that tattoos increase the risk of skin cancers [44–46], though a recent survey-based study of individuals aged 20–60 in the Swedish National Cancer Register associated tattoos with a 21% elevated risk of malignant lymphoma [47]. Patients are advised to weigh the benefits against the risks when considering decisions about getting tattoos.

Recent research has introduced a number of smart tattoos that are capable of optical sensing [48] and other functions. Subcutaneously injectable glucose biosensors have been in development for decades [49, 50], while intradermally implantable biosensors hold great promise for less invasive continuous physiological monitoring [51]. A number of biosensing smart tattoos have been demonstrated in *ex vivo* porcine skin models, where changes in tattoo color [52, 53] or fluorescence [54] can be correlated with changes in interstitial fluid analytes such as sodium, glucose, pH [52], or proteins [53]. Recently, smart-tattoo biosensors for uracil, pH, glucose, and temperature have been delivered intradermally with *in vivo* animal models [55, 56], though the chemosensing smart tattoos only functioned *in vivo* for several days [55]. Photosensing smart tattoos have been developed for colorimetric intradermal ultraviolet radiometry [57] and dosimetry [58, 59], as well as gamma ray dosimetry [60]. Other fluorescent smart tattoos include made-to-fade biopsy site markers with controllable intradermal retention times [61] and quantum dot-based vaccine authentication tattoos delivered by microneedle patch [56, 62]. These emerging smart tattoos expand the functional scope of aesthetic and medical tattoos.

Among the myriad use cases for tattoos, there are many in which it may be advantageous to have the ability to “turn off” the tattoo by rendering it invisible. Such kinds of switchable tattoos could prevent the need for painful and expensive tattoo removal procedures when a tattoo is not desired either due to regret [63] (for body art) or trauma [64] (for medical tattoos). Moreover, the ability to erase and rewrite a tattoo could open new doors for dynamic and re-configurable body art and permanent makeup. These considerations motivated the development of Magic Ink, a photo-rewritable tattoo ink recently introduced commercially in the United States [65]. Here we describe our testing and characterization of Magic Ink and the outcomes of our first clinical test of safety and efficacy of Magic Ink in an IRB-approved human study.

## Results and discussion

We report the physical characterization and preclinical testing of Magic Ink, the photochromic properties of Magic Ink, the clinical study design and results, and a colorimetric analysis of fully healed Magic Ink tattoos in both the ‘off’ and ‘on’ states.

### Preclinical testing and characterization of Magic Ink

**Fig. 1:**
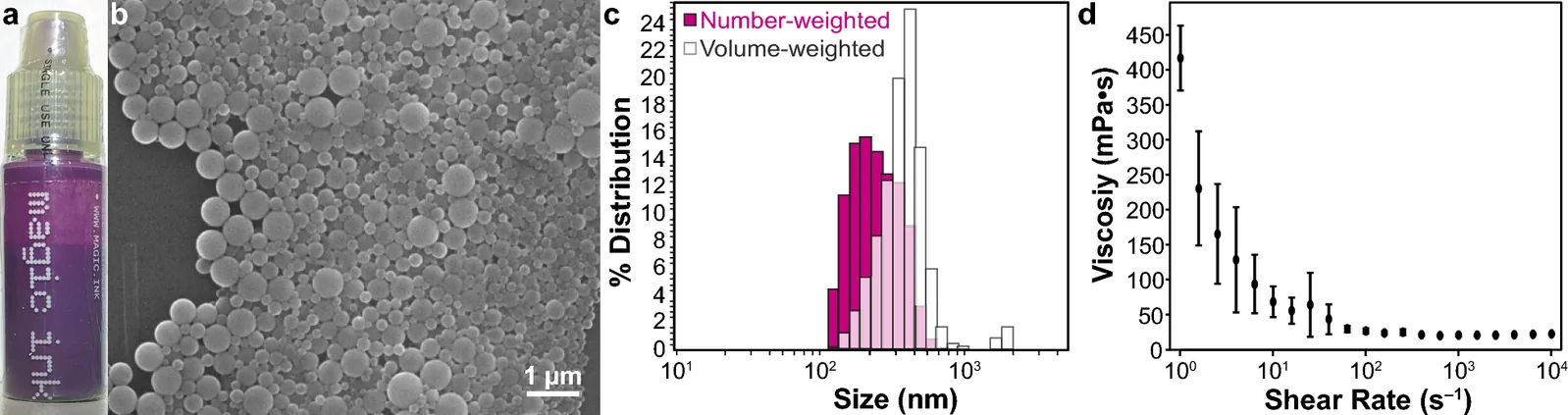
Characterization of Magic Ink. (**a**) Photograph of a bottle of Magic Ink. (**b**) Scanning electron micrograph of Magic Ink photochromic pigment particles. (**c**) Number- and volume-weighted size distributions of Magic Ink pigment obtained by dynamic light scattering. (**d**) Viscosity of Magic Ink as a function of shear rate. Error bars denote the standard deviation of the mean averaged over 3 trials

Magic Ink pigments comprise medical-grade PMMA particles in suspension stabilized by trace polyvinyl alcohol (<50 ppm). The pigment particles are doped with P-type photochromic diarylethene compounds [66, 67], which are responsible for the light-responsive properties of the tattoo pigment. [59] Magic Ink (Fig. 1a) was obtained from HYPRSKN Inc.; the Material Safety Data Sheet (MSDS) is included in the Supplementary Information. Magic Ink was characterized preclinically with regard to pigment size and morphology, shear-dependent viscosity, as well as third-party tests of purity, sterility, and toxicity.

#### Particle size and morphology

At least 50 different chemical compounds are in regular use as tattoo pigments in the US [68] and >100 pigments are employed in tattooing internationally, with at least 72 different tattoo ink brands [69, 70]. Tattoo pigments are typically sourced from industrial suppliers [71] and milled down to a desired size, which generally falls in the range of tens of nanometers to a couple of microns in diameter [72, 73]. Unlike the conventional process, which yields diverse pigment shapes and compositions, Magic Ink pigments are manufactured from high-purity, medical-grade materials by an emulsion-based nano-encapsulation process that yields uniformly spherical particles, as evidenced by scanning electron microscopy (SEM, Fig. 1b). Particle size analysis by dynamic light scattering (DLS) revealed a size distribution within the range of 100–1000 nm diameter (Fig. 1c), consistent with conventional tattoo inks, with a number-weighted average diameter of 235 nm, a volume-weighted average diameter of 386 nm, and a polydispersity index (PDI) of 0.099. The full set of parameters and data obtained by particle size analysis is included in Figure S1 of the Supplementary Information.

#### Rheological properties of Magic Ink

The flow properties of tattoo ink are optimized for efficient transfer of pigment into skin [74, 75], such that tattoo inks are shear thinning, with relatively low viscosities under shear. In our previous work [57], we found that commercial tattoo inks exhibit viscosities in the range of 10–1000 mPa*·*s. Presumably these good flow properties support the rapid transfer of ink into the voids created by the tattoo needle under high shear [76, 77]. Thus, we characterized the shear-dependent viscosity of Magic Ink by steady-shear rotational rheometry to verify that the viscosity and shear thinning behavior is consistent with conventional tattoo inks. Indeed, the flow curve in Fig. 1d shows that the viscosity of Magic Ink decreases from 417 ± 46 mPa*·*s at a shear rate of 1 s$^{-1}$ to 23 ± 1 mPa*·*s at shear rates up to 10$^{4}$ s$^{-1}$, confirming pronounced shear-thinning behavior. The rheological behavior of Magic Ink is thus comparable to that of conventional tattoo inks.

#### Sterility of Magic Ink

While bacterial infections in professional settings have become exceedingly rare [78], bacterial contamination can occur in any water-based ink, occasionally leading to major ink recalls by the FDA [79]. A 2013 survey of 58 unopened tattoo ink bottles from different manufacturers found bacterial contamination in 10% of the inks [80]. In 2018 a survey of 85 unopened tattoo inks from 13 US manufacturers found microorganisms in 49% of the inks (39% bacterial) [81]. The latter findings were confirmed again in 2020 [82]. The contamination risk is easily mitigated by verifying that the inks are sterilized before use. The standard for ink sterilization in the tattoo industry is gamma radiation because it is compatible with most materials and suitable for liquids in impermeable packages [83]. Therefore, the single-use 2-mL bottles of Magic Ink employed in this study (Fig. 1a) were sterilized with a dose of 25 kGy of gamma radiation (Steris, Libertyville, IL, USA). Zero out of five bottles selected randomly from the batch tested positive for microbial contamination in a sterility test performed at an accredited lab (Steris Libertyville North Laboratory) in compliance with the ANSI/AAMI/ISO 11737-2 and USP 71 standards of sterility testing [83]. The certificate of this third-party sterility testing is included in the Supplementary Information.

#### Purity testing of Magic Ink

Several families of chemicals are suspected to be problematic for tattoo inks owing to their known toxicity and/or carcinogenicity, including aromatic amines and polycyclic aromatic hydrocarbons. While different countries regulate contamination of tattoo inks differently, among the highest standards worldwide is the Registration, Evaluation, Authorization and restriction of CHemicals (REACH) regulation, which restricted over 4000 substances from tattoo inks in the European Union (EU) [84]. Recent research has shown that incorrect labeling and banned substances remain commonplace in the US [85] and EU [69]. Multiple surveys of tattoo inks [86, 87] have found that over half of commercial inks exceed the safe allergological limits for metals like Cr, Ni, and Co, which increase the risk of allergenicity [88]. Other impurities that have been found in tattoo inks include dibutyl phthalate (genotoxic, teratogenic), hexachloro-1,3-butadiene (genotoxic, carcinogenic), dibenzofuran, hexamethylenetetramine (possibly genotoxic), benzophenone (possibly carcinogenic), and 9-fluorenone [89]. The concerns may be linked to the fact that tattoo pigments are typically appropriated from industrial manufacturing processes and are of technical grade [71], with purities often less than 90% [90]. By contrast, the components of Magic Ink pigment are REACH-compliant and estimated by their manufacturers to be >99% pure. To verify the absence of potentially harmful impurities in Magic Ink, we submitted samples of Magic Ink for third-party testing (TIL Labs, Italy) of 13 metals and 31 aromatic amines banned in Europe under REACH regulations, which is a standard battery of tests performed for evaluating the safety of tattoo inks in the EU. The third-party testing report may be found in the Supplementary Information. As expected, none of these potentially harmful substances were detected above REACH-allowable limits in Magic Ink.

#### Effect of gamma sterilization on formaldehyde contamination

It is well known that gamma sterilization of tattoo ink generates formaldehyde [91], a reactive gas that, despite being naturally produced in metabolic processes of most living organisms, is toxic at sufficiently high concentrations and classified as a human carcinogen by the International Agency for Research on Cancer. While formaldehyde is not banned from cosmetics in the United States, the EU REACH regulations prohibit formaldehyde levels from exceeding 1 ppm in tattoo ink, presenting a challenge for inks sterilized by gamma radiation [91]. In bottles of Magic Ink sterilized with 25 kGy of gamma radiation, the formaldehyde content was observed to be 18.6 ppm (see the third-party purity testing report in Supplementary Information). We verified that sterilization was the sole source of formaldehyde contamination, since samples of Magic Ink withheld from sterilization did not test positive for formaldehyde, while bottles treated with a lower gamma dose of 1 kGy exhibited lower, but still detectable levels (>1 ppm) of formaldehyde. Despite exceeding REACH concentration limits, the absolute mass of formaldehyde delivered per tattoo is very low. Previous research [41, 77] has found that typical tattooing conditions yield intradermal pigment loadings on the order of *∼*1 mg/cm$^{2}$. The corresponding volume of deposited ink depends on its pigment load; assuming a conservative lower bound of 10 wt% pigment (the true value being higher; the pigment content of conventional tattoo inks is up to *∼*60 wt% [69]), this pigment dose corresponds to at most *∼*10 *μ*L of intradermal ink per square centimeter of skin. At our measured formaldehyde concentration of 18.6 ppm, the estimated dose is therefore <0.2 *μ*g per square centimeter of tattooed skin. Thus even large tattoos spanning tens of square centimeters represent a local formaldehyde dose in the low-*μ*g range. By comparison, steady-state concentrations of endogenous formaldehyde are 2.6 mg/L (blood) to 12 mg/L (intracellular) [92], and even inhalation at the OSHA-permissible 0.75 ppm exposure limit [93] is on the order of *∼*10 mg over an 8-hour workday, although direct comparisons are limited by differences in exposure route and pharmacokinetics. Nevertheless, mitigation of formaldehyde formation during sterilization remains an important target for further process optimization that will be required to achieve full global regulatory compliance.

#### Cytotoxicity testing of Magic Ink

Before the clinical study began, the photochromic dopant alone (not encapsulated) was sent for third-party cytotoxicity testing at Nelson Labs. This test was motivated by precaution against the unlikely case of photochromic dopants leaching from PMMA and migrating through cells or tissues. Leaching is unexpected because the dopant is completely insoluble in water; the long-term intradermal retention of Magic Ink observed and reported below also corroborates minimal loss of dye from the particles. Nevertheless, these precautionary tests ensure the dopant material would be non-toxic if it did escape from the host particles. For the cytotoxicity tests, extracts of the photochromic dopant and control materials were prepared in 1X Minimal Essential Media with 5% bovine serum (1XMEM5) and evaluated for *in vitro* cytotoxicity using a quantitative 3-(4,5-dimethylthiazol-2-yl)−2,5-diphenyltetrazolium bromide (MTT) optical assay in L929 mouse fibroblast cultures incubated for 24 h compliant with ISO 10993-5. Cultures exposed to the undiluted extract exhibited 74% cell viability, with increasing viability observed upon dilution, up to 86% viability with the 12.5% extracts. Because viability remained *≥*70% of the media control across all conditions, the photochromic material did not demonstrate cytotoxic potential under the conditions of this assay. Since PMMA has a long track record [94] of being successfully implanted in many tissues including skin [95], we did not initially consider a cytotoxicity test of the PMMA-encapsulated photochromic material, which is Magic Ink pigment itself. However, following the conclusion of the clinical study and commercial release of Magic Ink in the United States, we submitted a sample of Magic Ink pigment drawn from a commercially sold batch of Magic Ink for the same third-party cytotoxicity assay. The cell cultures exposed to undiluted extracts of Magic Ink pigment showed 91% viability, while all dilutions of the extract resulted in cell cultures that exceeded 100% viability. Thus, third-party testing confirmed that the PMMA-based pigment and photochromic dopants in Magic Ink are non-cytotoxic. Both the preclinical and post-commercial third-party cytotoxicity reports are included in the Supplementary Information.

#### Photochromic properties of Magic Ink

**Fig. 2:**
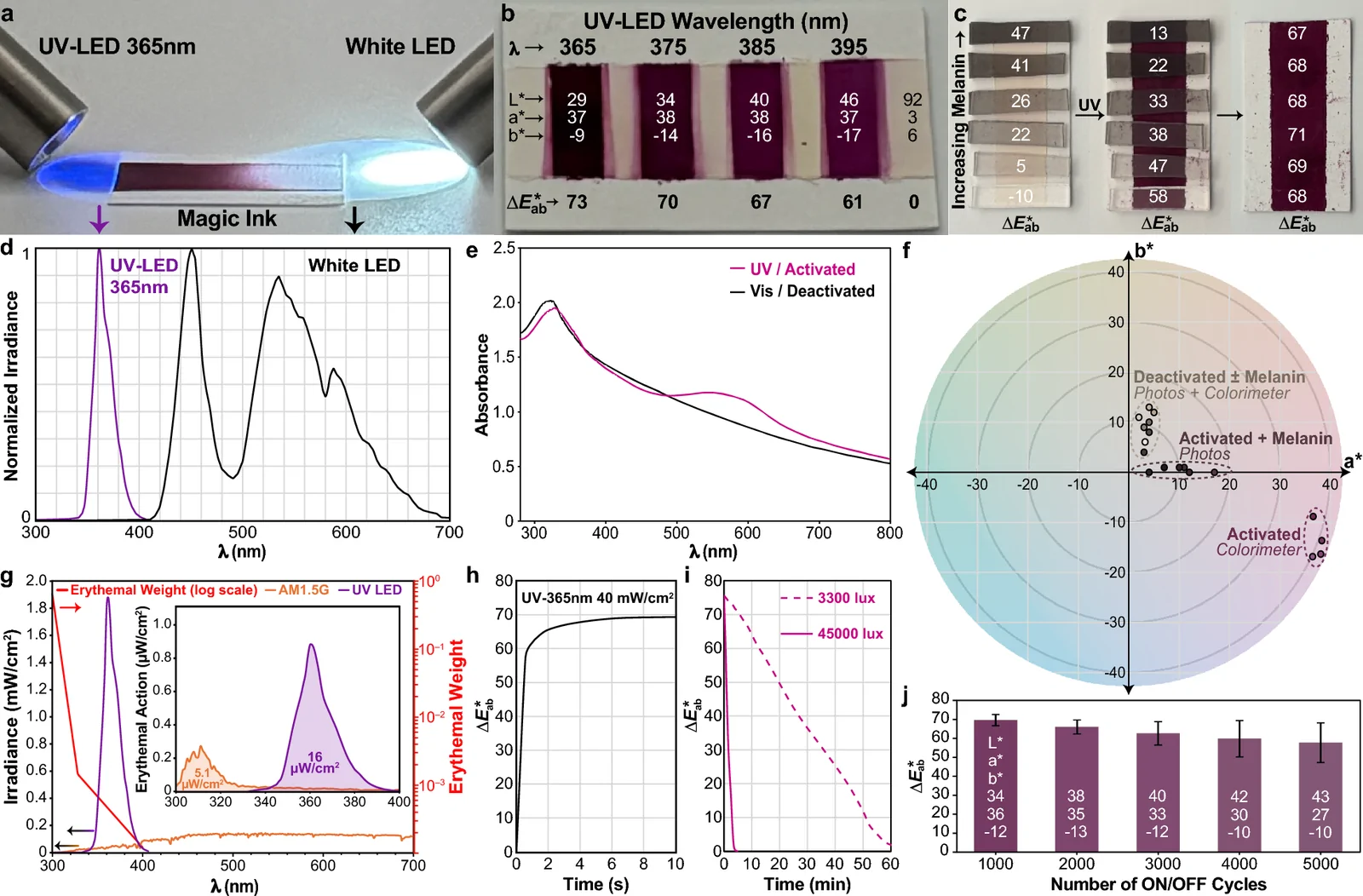
Photochromic characterization of Magic Ink. Panels (**a**–**f**) characterize the optical and colorimetric properties of the pigment, and panels (**g**–**j**) characterize its photoresponse to ultraviolet and visible light exposure. (**a**) Magic Ink paper swatch after exposure to UV and white LEDs. (**b**) CIELAB colorimetric readings (L*, black–white; a*, red–green; b*, blue–yellow) of Magic Ink pigment in the photostationary state under 365, 375, 385, and 395 nm UV-LED light; the $\Delta E_{ab}^{*}$ value above each section is the chromaticity difference between the UV-activated and deactivated states at that wavelength. (**c**) Effect of melanin-doped silicone skins on the chromaticity of Magic Ink; the $\Delta E_{ab}^{*}$ values in the two outer photographs are referenced to bare deactivated pigment, while those in the central photograph are referenced to melanin-coated deactivated pigment from the same swatch section. (**d**) Spectral power distributions of the 365 nm UV-LED and the blue-pumped white LED packaged with Magic Ink. (**e**) Absorbance spectra of a dilute Magic Ink suspension before (black) and after (magenta) 365 nm UV-LED exposure. (**f**) Projection of the colorimetric data from panels (**b**) and (**c**) onto the a*–b* plane of CIELAB space; each point is rendered in the RGB color corresponding to its L*-a*-b* values. (**g**) AM1.5G solar (orange) and UV-LED (violet) irradiance shown together with the erythemal weighting function (red, logarithmic scale); the inset shows the erythemal action spectrum of each source. (**h**) UV activation kinetics: $\Delta E_{ab}^{*}$ versus irradiation time under a 365 nm UV-LED at 40 mW/cm$^{2}$. (**i**) White-light deactivation kinetics: $\Delta E_{ab}^{*}$ versus irradiation time under white LEDs at 3300 and 45000 lux. (**j**) Fatigue resistance: average colorimetric values of Magic Ink swatches after 1000–5000 UV/white (ON/OFF) switching cycles. Column colors encode the inscribed L*-a*-b* values in RGB and error bars denote the standard deviation of the mean averaged over 9 readings of 3 samples

 The photochromic and colorimetric properties of Magic Ink are summarized in Fig. 2. Thin films were deposited on Bristol paper by doctor blading to analyze the properties of the bare Magic Ink pigment in response to ultraviolet and visible light (Fig. 2a). We recorded colorimetric readings of these paper Magic Ink swatches using both digital photography and a colorimeter. We report these colorimetric readings in CIELAB chromaticity space [96], which defines color along black-white (L*), red-green (a*), and blue-yellow (b*) axes. We selected this color system not only because it is widely used in photochromic materials research [97], but also because Magic Ink is designed for human use and CIELAB is calibrated to the human visual system; it was constructed so that a color difference (labeled $\Delta E_{ab}^{*}$, the Euclidean distance between two colors in CIELAB chromaticity space) of magnitude *∼*1 or greater corresponds to a just-noticeable difference (JND) [98].

#### UV wavelength dependence of photochromic response

The coloration of the Magic Ink swatch after prolonged exposure to four different UV LEDs (365–395 nm) is presented in Fig. 2b. Each colored section of the swatch was irradiated with a UV LED for 60 s to ensure full population of the photostationary state (PSS). The L*-a*-b* values inscribed within each section of the swatch were obtained using a colorimeter, while the $\Delta E_{ab}^{*}$ values indicate the magnitude of color difference between the UV-activated area and the deactivated colorless/pale-beige background color of Magic Ink. The UV-induced coloration becomes weaker with increasing excitation wavelength, from a maximum of $\Delta E_{ab}^{*}$=73 at 365 nm to a minimum of $\Delta E_{ab}^{*}$=61 at 395 nm. The diminished photochromic response at longer wavelengths is attributable to poor absorption of the photochromic dopant at these longer wavelengths. Thus, although Magic Ink is responsive to all UV wavelengths, we selected 365 nm as the ideal UV-LED for Magic Ink activation owing to its optimal balance of strong activation, low cost, and relatively low erythemal weights (discussed below) for the sake of skin safety. The spectral power distribution of the 365 nm UV-LED light packaged with Magic Ink is compared with that of the broad-spectrum blue-pumped white LED used for deactivation in Fig. 2d.

#### Effect of melanin on the appearance of Magic Ink

In previous work [59] we employed melanin-doped artificial skins made of silicone to model how photochromic tattoo pigments would behave under lighting that is filtered through melanin, as in real human skin *in vivo*. Fig. 2c displays a sequence of photographs from an experiment where these artificial skins were placed on top of a Magic Ink paper swatch, fully activated with 365 nm UV-LED light, and then analyzed colorimetrically from the photographs with and without the melanated skins. Placing these silicone filters on top of Magic Ink leads to a change in the baseline color of the deactivated pigment, shifting toward the color of the artificial skin itself. The skins with low melanin content showed minor differences with the bare pigment of $\Delta E_{ab}^{*}$=5–10, while the more highly melanated samples drift as far as 47 steps away from bare pigment color. Naturally, this domination of the color profile by melanin also affects the appearance of the UV-activated Magic Ink pigment. Although the pigment itself becomes equally activated ($\Delta E_{ab}^{*}$ 67–71) underneath all melanin filters, melanin’s own color diminishes the contrast between the skin and the pigment, such that perceptibility drops from $\Delta E_{ab}^{*}$=57 to $\Delta E_{ab}^{*}$=13 as seen (Fig. 2c) through skins with increasing melanin content. This result indicates that Magic Ink can be activated in all skin tones but becomes more difficult to differentiate from the background skin tone with increasing melanin content, consistent with the behavior of ordinary tattoo inks [99].

#### Colorimetric analysis of Magic Ink performance

The colorimetric readings from the UV-activation experiments described above are projected onto the a*–b* plane of CIELAB chromaticity space in Fig. 2f. This projection ignores the substantial differences in lightness/darkness encoded by L*, visualizing only differences in chromatic hue defined by the a* and b* axes. All of the deactivated Magic Ink readings, regardless of melanin content, are clustered together in a region with low a* values (3–5) reflecting the pigment’s low visible absorption, and more positive b* values (4–12) reflecting the slight pale-beige color of both deactivated Magic Ink pigment and melanin. Activation with UV light causes b* to decrease and a* to increase; the UV-activated samples observed through melanin are clustered at neutral b* values of 0–1 and moderately positive a* values of 4–17, making them appear red-brown. The uncoated bare Magic Ink pigment appears more magenta-purple, with negative b* values (–9––17) and highly positive a* values of 37–38. The magenta color that develops when Magic Ink is exposed to UV light is attributable to a reversible photo-isomerization reaction that causes a new visible absorption band ($\lambda _{max}$ = 545 nm) to emerge in the UV-Vis spectrum (Fig. 2e) of the material. These spectra obtained from a dilute suspension of Magic Ink show typical features of Rayleigh scattering, attributable to small particles with diameters on the same size scale as the wavelengths of visible light, with a sloping baseline reflecting diminished scattering at longer wavelengths.

#### Comparison of solar and UV-LED radiation

The irradiance of the UV-LED packaged with Magic Ink is compared with the Air Mass 1.5 Global (AM1.5G) solar spectrum [100], representing an average sunny day: mid-latitude clear-sky conditions of sunlight passing through 1.5x atmosphere (48.2$^\circ$ zenith angle) at 37$^\circ$ tilt, in Fig. 2g. The AM1.5G solar spectrum contains *∼*4 mW/cm$^{2}$ total UV (280–400nm) radiant power, which is ten-fold lower than the *∼*40 mW/cm$^{2}$ total UV flux recorded for the UV-LED at a distance of 4 cm. Overlaid on these two spectra in Fig. 2g is the erythemal weighting function (in log scale to support visualization), which adjusts raw UV measurements to reflect that shorter wavelengths (UVB, 280–320 nm) are far more damaging to skin than longer ones (UVA, 320–400 nm), based on the CIE McKinlay-Diffey action spectrum [101]. Thus the erythemal-weighted irradiance of average sunlight and the UV-LED, obtained as the product of irradiance and erythemal weight at each wavelength, is inset in Fig. 2g. The area under this erythemal action spectrum is only *∼*3-fold greater for the UV-LED (16 *μ*W/cm$^{2}$) than AM1.5G solar radiation (5.1 *μ*W/cm$^{2}$), since unlike sunshine the UV-LED does not output UV light below 330 nm where erythemal weights [101] increase more severely. We recommend avoiding UV-LED sources of $\lambda _{max}<$365 nm to activate Magic Ink in order to avoid exposing skin to these much more harmful wavelengths of UV light ($\lambda _{UV}\le$330 nm) contained in sunshine.

#### Kinetics of UV activation

The photochromic properties of Magic Ink pigment have been optimized to be highly sensitive to UV light; short exposure times minimize risk to skin and maximize the rapid UV-activation of the tattoo. As shown in Fig. 2h, a Magic Ink paper swatch irradiated with the UV-LED light at 4-cm distance (*∼*40 mW/cm$^{2}$) becomes completely activated in seconds. The sample reached 88% maximum activation ($\Delta E_{ab}^{*})_{avg}$ = 62) at 1 s, 93% at 2 s ($\Delta E_{ab}^{*})_{avg}$ = 65), and 94% at 3 s ($\Delta E_{ab}^{*})_{avg}$ = 67). Given the rapidly diminishing returns, no more than 2–3 s of exposure time is needed, since further differences in coloration beyond 3 s of exposure are near the lower limit of human perception.

#### The UV dose for Magic Ink activation

Based on the irradiance and kinetics measurements described above, the UV dose associated with activating Magic Ink can be estimated and compared (Fig. 2g) with that of solar light on an average sunny day. Since the radiant flux of UV light in the UV-LED is 10-fold greater than that of AM1.5G radiation, the total dose of UV light administered in the 2-second exposure required to activate Magic Ink to roughly 93% of its maximum coloration is equivalent to that acquired in *∼* 20 s of average sunlight exposure. However, since AM1.5G sunlight contains UVB light which the UV-LED lacks, the erythemal-weighted dose associated with this 2 s exposure is equivalent to only *∼*6 s of average sunlight. At the UV-LED’s power density of 16 *μ*W/cm$^{2}$, it would take 10 minutes and 25 seconds to reach a standard erythemal dose (SED), defined [102] as 100 J/m$^{2}$. Since even the most photosensitive, melanin-depleted skin types (Fitzpatrick I–II) require [103] 2–2.5 SED to reach a minimum erythemal dose (MED, the minimum dose required to generate observable skin redness after 24 h), in principle Magic Ink could be activated up to several hundred times per day without causing erythema, assuming minimal exposure times required to reach *∼*93–94% activation are maintained through a transparent epidermis. This estimation represents an upper limit, since UV absorption in the epidermis will attenuate the UV light incident on dermis, lengthening the required exposure time (increasing as melanin content and/or epidermal thickness increases), thus reducing the number of activations that are possible without inducing erythema depending on skin tone and anatomical location of the tattoo.

#### Kinetics of white light deactivation

Since the photo-isomerization of P-type photochromic compounds is thermally irreversible [66], the color of Magic Ink is retained long after UV exposure ceases; deactivation of Magic Ink can only be achieved by exposure to sufficient visible light. Magic Ink was designed to be relatively insensitive to visible light, such that ambient indoor lighting is not sufficient to deactivate Magic Ink on the timescale of at least several hours. For example, the decoloration of UV-activated Magic Ink is plotted as a function of time in two lighting scenarios in Fig. 2i. At 3300 lux (which is at least 3x brighter than most indoor lighting conditions, on par with an overcast day) the Magic Ink paper swatch requires *∼*1 hour to fully decolorize, but at 45000 lux (white LED at 4 cm), the decoloration time is reduced to under 5 minutes (Fig. 2i). Bringing the white LED in contact with skin or exposing Magic Ink to direct sunlight filtered through topical sunscreen can exceed 100000 lux, where deactivation is complete in tens of seconds. This high UV sensitivity and low visible sensitivity is what underlies the rewritability of Magic Ink in the context of body art or medical tattoos, allowing the photo-activated state of the tattoo to persist for hours to days in shade and indoors, yet erase the design rapidly under direct illumination of bright visible light.

#### Fatigue resistance of Magic Ink

An important but often overlooked aspect of photochromic materials is fatigue resistance, the ability to undergo many activation-deactivation cycles without degrading into non-photochromic byproducts. Diarylethenes have well-known capacity for good fatigue resistance [104], but in fact their fatigue properties vary widely from poor to excellent depending on both compound structure [105, 106] and the absorbance of the ring-closed isomer at the wavelength(s) of UV irradiation [107]. The colorimetric data summarized in Fig. 2j demonstrates that Magic Ink exhibits excellent fatigue resistance at 365 nm irradiation, since it can undergo thousands of photoswitching cycles with minimal losses. In the fatigue experiment, the Magic Ink paper swatch began with a $(\Delta E_{ab}^{*})_{avg}$ value of 71 on the first UV activation, which reduced only to $(\Delta E_{ab}^{*})_{avg}$ = 70 after the first thousand ON-OFF cycles. The magnitude of $(\Delta E_{ab}^{*})_{avg}$ decreased linearly until $(\Delta E_{ab}^{*})_{avg}$ was 58 after 5000 cycles. The standard deviation of the mean (denoted by error bars in Fig. 2j) increased over repeated cycles because more photo-degradation occurred at the center of the swatch positioned closer to the UV-LED light source. It is noteworthy that each UV activation cycle involved a 15 s exposure, far longer than required, which further underscores the high fatigue resistance of Magic Ink pigment at an excitation wavelength of 365 nm.

### Clinical study of Magic Ink

The purpose of the clinical study was to compare the healing behavior of Magic Ink and Standard Ink tattoos in human subjects with respect to safety (absence of adverse events and major clinical abnormalities) and tolerability (participant- and dermatologist-reported symptoms). Immediately after receiving a tattoo, the skin’s typical healing course begins with an acute inflammatory response. Some pigments are excreted through the epidermis by desquamation and transepidermal elimination, retaining only intradermal pigments over *∼*1 month of healing [108].

In the dermis, melanophages are recruited to the wound site to engulf the tattoo pigments. A minority of these melanophages then migrate to deposit pigment in the lymph nodes [109–111], most of which are less than 200 nanometers in diameter [112]. There is also evidence that intradermally and subcutaneously injected nanoparticles (quantum dots [113], silver [114], and fluorescent polymers [61]) can be deposited in other organs such as the liver, kidney, and spleen in rodents [115]. One investigation reported a 32% decrease in local tattoo pigment concentration over a six-week healing period in a murine tattoo model [33]. The majority of pigments remain intradermal long-term, undergoing repeated cycles of capture and release from these immune cells [116]. By tracking Magic Ink tattoos systematically over a long period of time, we aimed to support the expectation that Magic Ink mirrors the normal healing behavior and permanent retention of conventional tattoo inks.

#### Clinical study design

**Fig. 3:**
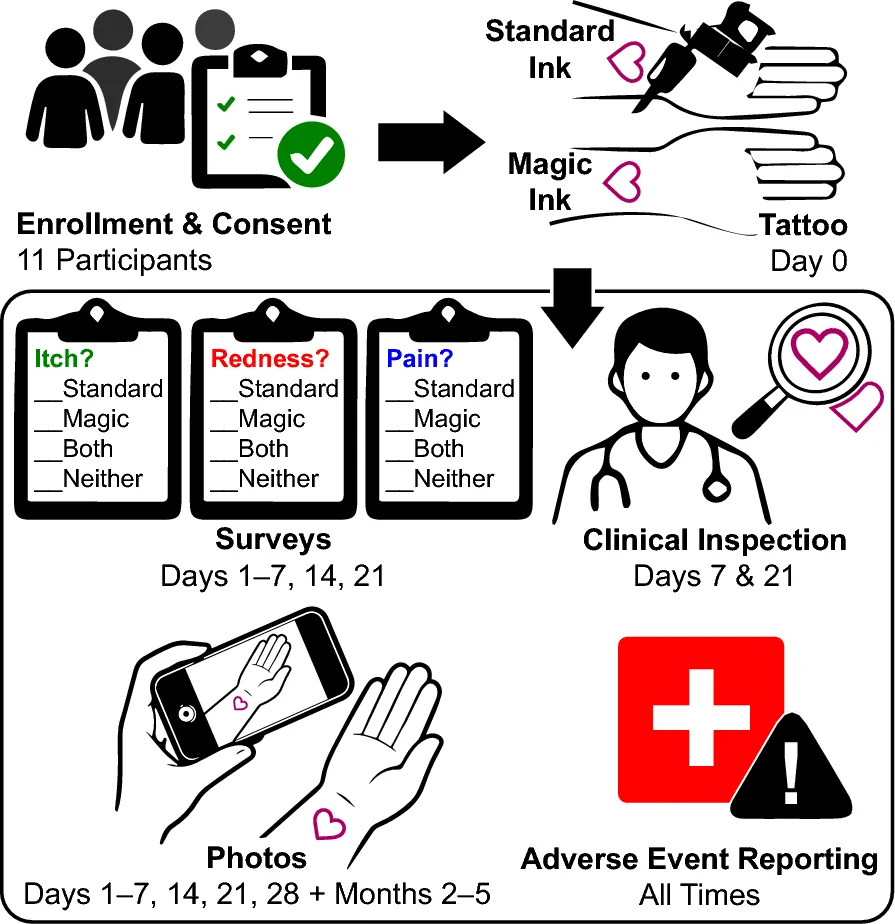
Overview of clinical study design comparing the healing course of Magic Ink tattoos with color-matched control tattoos made with commercially available ’Standard Ink’

 The IRB-approved clinical study was designed (Fig. 3) to compare the healing profile of Magic Ink directly with a well-established commercial tattoo pigment to evaluate its safety and tolerability in human skin. Eleven participants were recruited to the study with informed consent. Participants were recruited by purposive sampling from the clientele of the licensed tattoo artist who performed the procedures, ensuring a study population with prior tattoo experience consistent with the inclusion criteria. The participants included two females and nine males, spanning a wide range of skin tones (Fitzpatrick types I–V) and an age range of 28–62 years (Table 1), thus capturing a diverse range of skin qualities. As an inclusion criterion, all participants had received prior tattoos without adverse reactions, thus excluding patients with general tattoo hypersensitivities. Each participant received two identical heart-shaped tattoos placed symmetrically on opposing appendage sites, one with Magic Ink and one color-matched control with Starbrite Deep Burgundy tattoo ink (‘Standard Ink’). For example, if a participant selected the ankle for the tattoo site, they would receive one Standard Ink tattoo on the right ankle, and one Magic Ink tattoo on the left ankle, or *vice versa*. For the sake of consistency, all tattooing was performed by one licensed tattoo artist with >10 yrs experience, who was instructed to aim for consistent procedural treatment across all inks and participants. After the procedure, participants were given aftercare instructions and ointment (Figure S5, Supplementary Information) and instructions on how to provide periodic survey feedback and photographs in order to support documentation of the healing process. The tattoo photographs were received and reviewed by a licensed, board-certified dermatologist and dermatopathologist at time intervals of 1 d, 2 d, 3 d, 4 d, 5 d, 6 d, 7 d, 14 d, 21 d, 28 d, 2 mo, 3 mo, 4 mo, and 5 mo. Both tattoo sites were inspected by the dermatologist in-person after 7 and 21 days for erythema, swelling, scale/crust, and tenderness. Survey responses were collected from the participants on days 0–7, 14, and 21. Participants answered three survey questions to report their experience of both tattoos with respect to itch, redness, and pain at each time interval.

**Table 1:** Participant summary

ID	Sex	Age	Skin tone	Tattoo site
P1	M	38	V	ankle
P2	M	43	II	shin
P3	M	62	II	ankle
P4	M	58	IV	wrist
P5	M	38	III	forearm
P6	M	52	IV	wrist
P7	M	36	II	wrist
P8	F	40	I	forearm
P9	F	32	I	bicep
P10	M	40	III	ankle
P11	M	28	III	ankle

#### Clinical safety assessment

As of this publication, no adverse events or abnormalities have been reported by any participants for any of the tattoos in three years since the clinical study began in Q1 2023. Adverse events were defined as any unexpected local or systemic reaction requiring medical intervention or deviating from the normal course of tattoo healing. Although no adverse events were observed (0/11 per group), the Clopper–Pearson 95% confidence interval allows for a true adverse event rate of up to 24% in both tattoo groups, given the limited sample size of this pilot study.

#### Clinical tolerability assessment

**Fig. 4:**
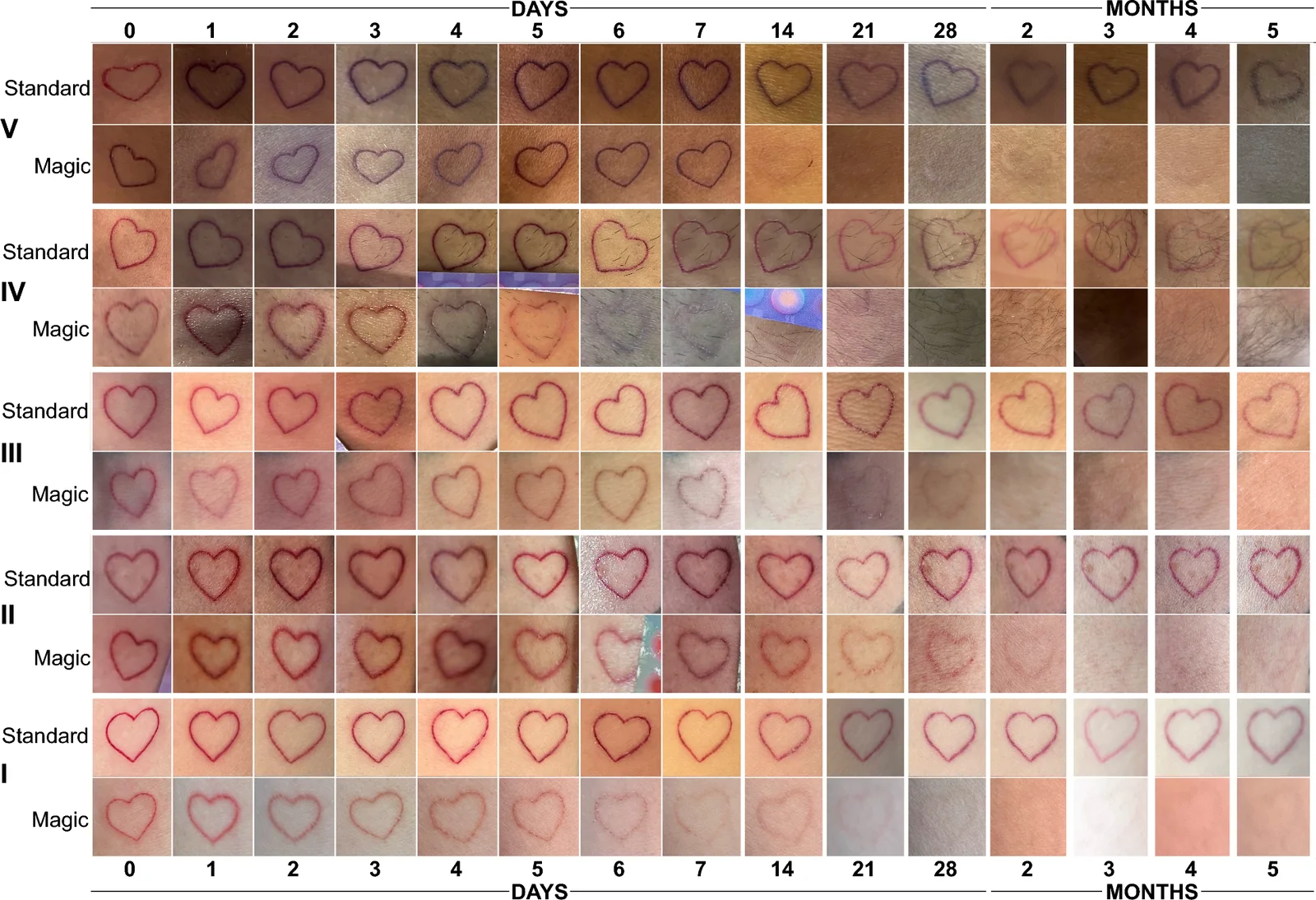
Photographic timelines of participant-submitted healing documentation comparing the appearance of Magic Ink and Standard Ink over a 5-month healing course for five representative skin tones/participants

**Fig. 5:**
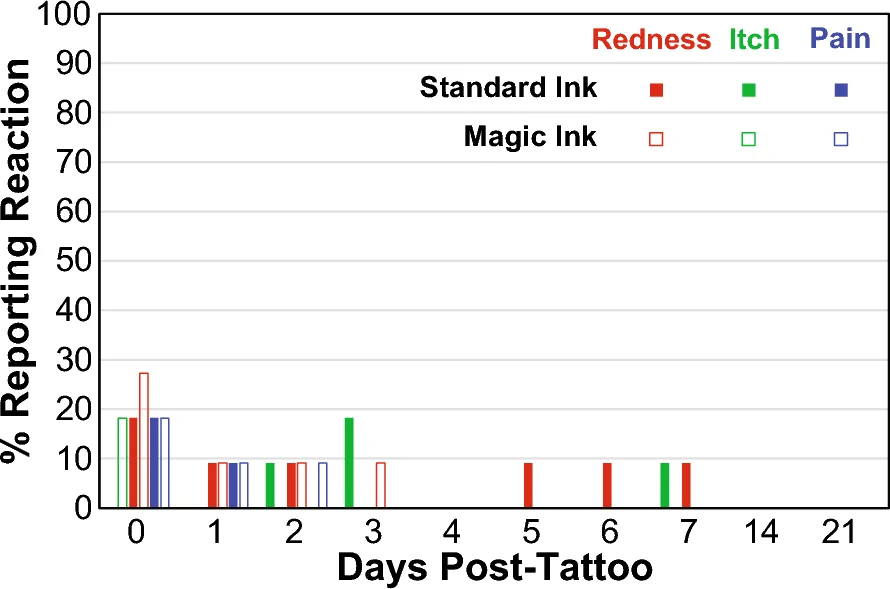
Summary of survey results collected at days 0–7, 14, and 21 indicating the percentage of respondents reporting redness, itch, or pain at each tattoo site

Clinical tolerability was assessed through both dermatologist evaluation and participant-reported outcomes. An evaluation by a board certified dermatologist was performed on days 7 and 21 evaluating for erythema, swelling, scale/crust, and tenderness. On day 7, 89% of the subjects had zero reaction at either tattoo site. Among the remaining 11% of patients, the only reaction identified was “scale/crust,” which was observed slightly more frequently at Magic Ink tattoo sites than Standard Ink tattoo sites. No abnormalities were noted for any of the tattoos during either in-person clinical inspection; scale/crust observed in 11% of patients at day 7 was well within the bounds of a normal tattoo healing course. By day 21, 100% of recipients had zero reaction at either tattoo site. The daily, weekly, and monthly photographic documentation (Fig. 4, Figures S2–S3 in Supplementary Information) is qualitatively consistent with the clinical observations and survey data. In the subject-reported survey data (Fig. 5), 94% of all responses indicated zero pain, redness, or itch at either the Magic Ink tattoo or Standard Ink tattoo sites. Of the remaining 6% of responses, the outcomes were equally distributed: 2% reported experiencing more pain/redness/itch at the Standard Ink tattoo site when compared to the Magic Ink tattoo site, 2% reported more pain/redness/itch at the Magic Ink tattoo site when compared to the Standard Ink tattoo site; 2% reported equal symptom burden at both tattoo sites. Given the low incidence of symptoms and small number of discordant paired observations, statistical comparisons were underpowered; exact McNemar tests showed no significant differences (p*>>*0.05) at any timepoint. Taken together, these findings indicate that healing outcomes were similar between Magic Ink and Standard Ink control tattoos across all measured endpoints.

### Colorimetric analysis of Magic Ink efficacy *in vivo*

In the context of Magic Ink, efficacy means long-term intradermal retention with sustained reversible photoswitching function and perceptibility of the tattoos *in vivo*. Collecting participant-submitted photographs afforded us an opportunity to perform colorimetric analysis of the tattoos. Participants were advised not to activate their Magic Ink tattoos with UV light until the conclusion of the 5-month healing course; thus the photographic timelines in Fig. 4 demonstrate that Magic Ink tattoos gradually become almost indistinguishable from the surrounding skin in the deactivated state, owing to their deactivation by ambient light.

#### Quantifying tattoo color in CIELAB chromaticity space

**Fig. 6:**
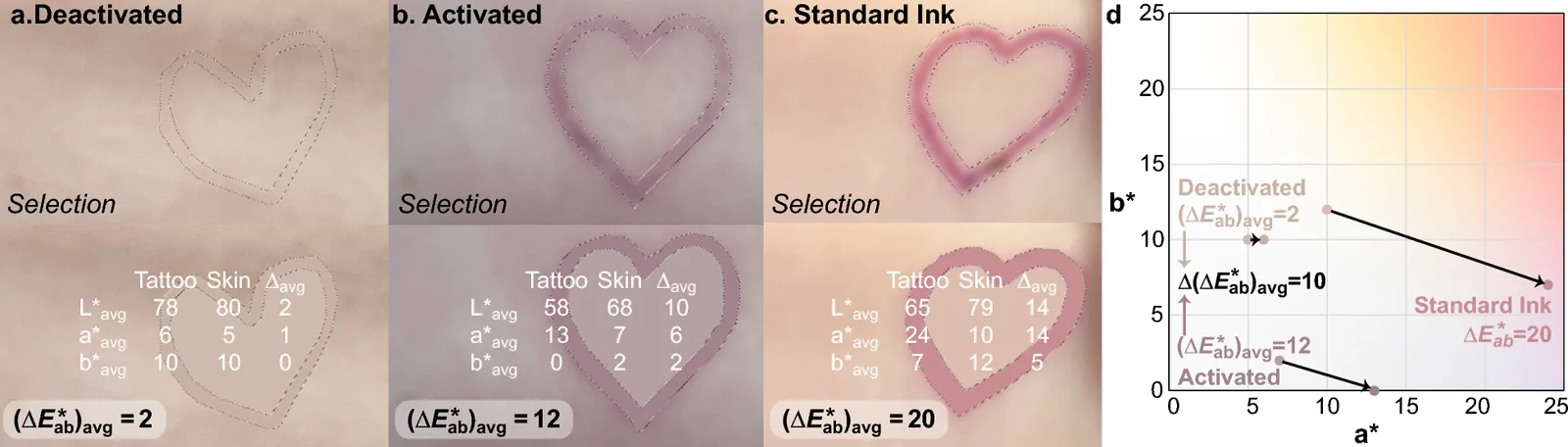
Demonstration of colorimetric comparison for tattoos after 5 months of healing. The average colors (L$^{*}$, a$^{*}$, and b$^{*}$ values in CIELAB chromaticity space) of the tattooed and non-tattooed skin were extracted from participant-submitted photographs for (**a**) Deactivated Magic Ink, (**b**) Activated Magic Ink (365 nm), and (**c**) Standard Ink tattoos in P8 after 5 months of healing. (**d**) The $(\Delta E_{ab}^{*})_{avg}$ values quantify the difference in color between the tattooed and non-tattooed skin for the deactivated and activated Magic Ink tattoos and the Standard Ink control tattoo

**Fig. 7:**
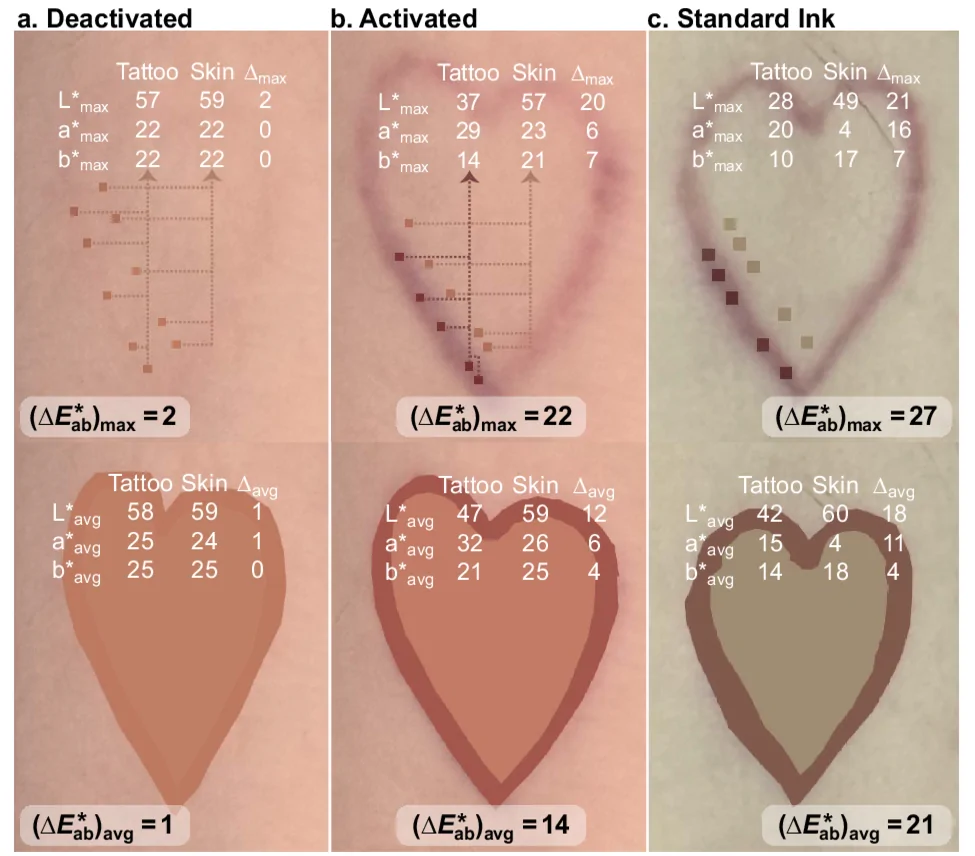
Comparison of methods for estimating $(\Delta E_{ab}^{*})_{max}$ and $(\Delta E_{ab}^{*})_{avg}$. The (**a**) deactivated and (**b**) UV-activated Magic Ink tattoos aged 12 months are compared with (**c**) the Standard Ink tattoo of P7 aged 5 months. $(\Delta E_{ab}^{*})_{max}$ is taken from the average difference in CIELAB chromaticity space of five 10x10 pixel grids sampled from the darkest areas of the tattoo and five equidistant 10x10 grids in the nearby bare skin. $(\Delta E_{ab}^{*})_{avg}$ compares these average CIELAB values of the entire tattoo area against the entire bare-skin area inside of the tattoo shape

Our procedure for quantifying the visual appearance of the Magic Ink and Standard Ink tattoos in participant-submitted photographs is demonstrated in Figs. 6–7. One participant activated their Magic Ink tattoo with the UV-LED light before submitting their final photograph, enabling one side-by-side comparison of Magic Ink and Standard Ink colorimetry at month 5 (Fig. 6). After UV activation, the Magic Ink tattoo appears substantially darker (L* 68*→*58), more red (a* 7*→*13), and slightly less yellow (b* 2*→*0), corresponding to a sixfold increase in color contrast with the background skin ($(\Delta E_{ab}^{*})_{avg}$ = 12). The same participant’s Standard Ink control exhibited a 67% greater average color difference ($(\Delta E_{ab}^{*})_{avg}$ = 20) with the background skin at the same time point. The UV-induced change in contrast of Magic Ink from $(\Delta E_{ab}^{*})_{avg}$ 2*→*12 is quantified simply by their difference in magnitude, $\Delta (\Delta E_{ab}^{*})_{avg}$ = 10 (Fig. 6d). The deactivated Magic Ink tattoo is difficult to locate in the participant-submitted photograph because the difference in color between the tattoo and adjacent skin is close to the lower limit of human perception ($(\Delta E_{ab}^{*})_{avg}$ = 2). Although a $\Delta E_{ab}^{*}$ value of 1 is a common heuristic for the JND [98], modern research has concluded that the JND is closer to $\Delta E_{ab}^{*}$=2.3 [117]. Thus, the visibility of deactivated Magic Ink compared to normal skin is at or below the JND on average. This near-invisibility in the off state gives the perceptual impression that the tattoo is ‘erased’ by white light.

#### Magic Ink efficacy one-year post-procedure

**Fig. 8:**
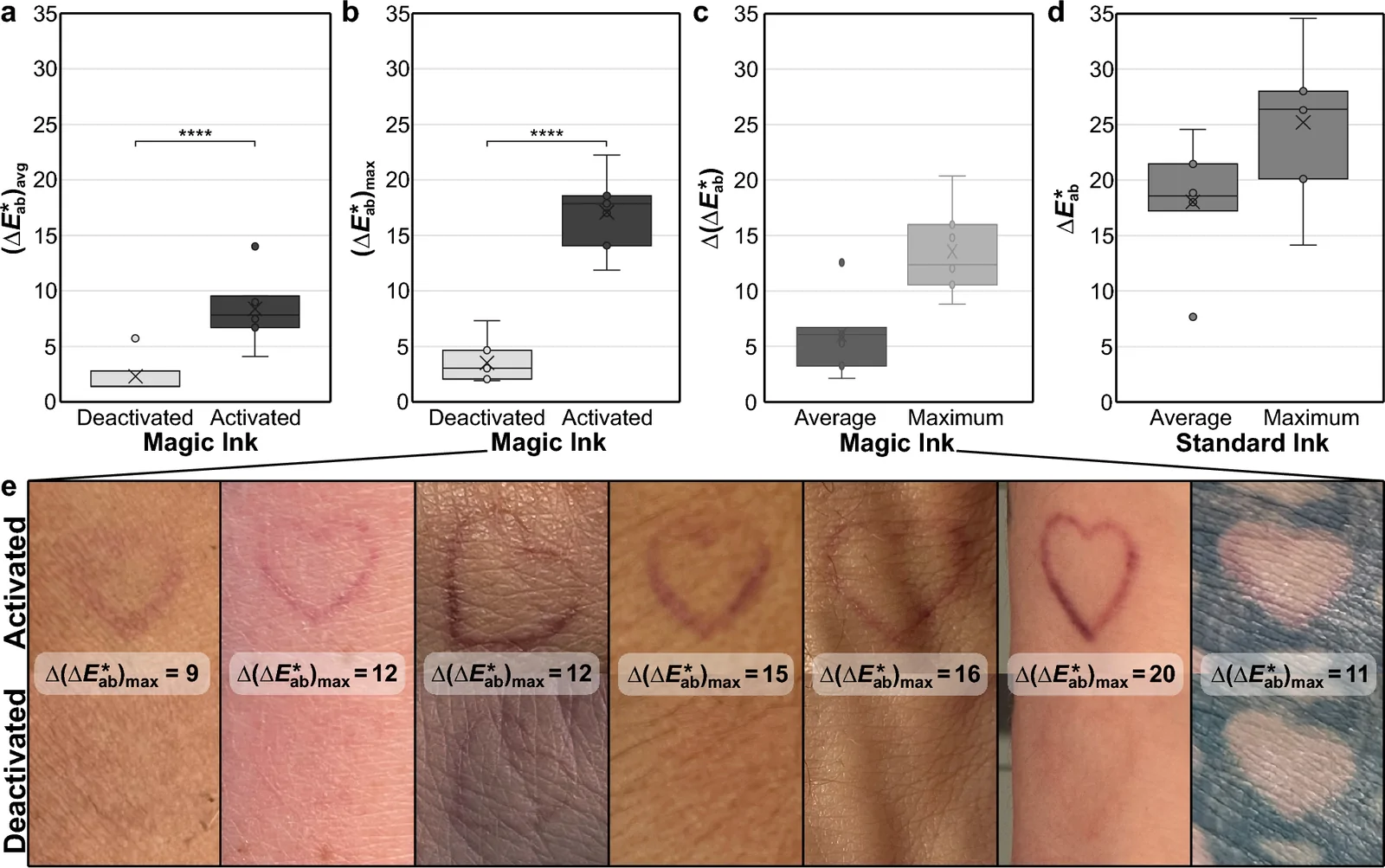
Quantitative colorimetry of Magic Ink at 12 months and Standard Ink at 5 months extracted from participant-submitted photographs, including box plots of (**a**) $(\Delta E_{ab}^{*})_{avg}$ (**b**) $(\Delta E_{ab}^{*})_{max}$ for Magic Ink in the deactivated and activated states, (**c**) average and maximum $\Delta (\Delta E_{ab}^{*})$ for Magic Ink and (**d**) average and maximum $(\Delta E_{ab}^{*})$ for Standard Ink relative to the background bare skin. (**e**) Seven pairs of photographs used for this colorimetric analysis annotated with $\Delta (\Delta E_{ab}^{*})_{max}$ values calculated for each pair

After the clinical study concluded, all participants were invited to optionally submit photographs of their activated and deactivated Magic Ink tattoo after 12 months. Seven participants submitted pairs of photos taken before and after local UV-LED exposure, though we could not verify correct illumination conditions because participants exposed and photographed their tattoos independently. Nevertheless, the photos still confirmed the expectation that Magic Ink tattoos are permanent, and they continue to function as photoswitchable pigments *in vivo* for at least one year. Fig. 7 demonstrates how we calculate $(\Delta E_{ab}^{*})_{max}$ by selecting and averaging five 10x10 pixel samples from the darkest parts of a tattoo against five equally sized samples of the nearby skin. The $(\Delta E_{ab}^{*})_{avg}$ and $(\Delta E_{ab}^{*})_{max}$ values represent colorimetric lower and upper bounds of Magic Ink’s visibility *in vivo* after one year. Both figures of merit are summarized for the seven participant-submitted pairs of Magic Ink tattoo photographs received after 12 months in Fig. 8 and Figure S4 of the Supplementary Information. Magic Ink tattoos are nearly imperceptible when deactivated, since their average $(\Delta E_{ab}^{*})_{avg}$ (2.3±1.6) and $(\Delta E_{ab}^{*})_{max}$ (3.5±1.9) values are close to the JND of 2.3 [117]. Magic Ink tattoos become significantly (*p<*0.00001 by two-tailed paired t-test, Fig. 8a-b) more visible than the deactivated tattoos with UV activation for both $(\Delta E_{ab}^{*})_{avg}$ (8.4±3.0) and $(\Delta E_{ab}^{*})_{max}$ (17.1±3.3), and these color changes are perceptible ($\Delta (\Delta E_{ab}^{*})_{avg}$ 6.0±3.3 and $\Delta (\Delta E_{ab}^{*})_{max}$ 13.6±3.8, Fig. 8c). However, activated Magic Ink tattoos at 12 months are only 46% (average) to 68% (maximum) as high in contrast as that of the Standard Ink tattoos (($\Delta E_{ab}^{*})_{avg}$ 18.1±5.2 and $(\Delta E_{ab}^{*})_{max}$ 25.2±6.4, Fig. 8d) observed at five months (unfortunately, we did not receive Standard Ink tattoo photographs at 12 months for comparison). In summary, colorimetric analysis of participant-submitted photographs demonstrates that Magic Ink tattoos are nearly imperceptible when deactivated and highly perceptible when activated by 365 nm UV light, but not as dark as a color-matched Standard Ink.

## Conclusions

This work evaluated Magic Ink, a photo-rewritable photochromic tattoo ink, through preclinical characterization and a pilot clinical comparison with a commercial control ink. Preclinical testing showed that the pigment switches reversibly between a near-colorless state and a magenta state, with UV activation reaching >90% of maximum coloration within seconds, controllable visible-light deactivation, and retention of photochromic response over thousands of switching cycles. Third-party testing established the sterility and purity of Magic Ink and found no cytotoxic potential for either the photochromic dopant or the PMMA-encapsulated pigment, while particle sizing and rheometry indicated a size distribution and shear-thinning behavior consistent with conventional tattoo inks. In the clinical study, 11 participants each received matched Magic Ink and Standard Ink tattoos on opposing appendage sites. No adverse events were observed in either group, dermatologist assessments identified no clinically meaningful abnormalities, and participant-reported pain, redness, and itch did not differ significantly between the two inks. Colorimetric analysis of participant-submitted photographs indicated that Magic Ink tattoos remained near the lower threshold of visual perception in the deactivated state, yet became readily visible upon UV activation in the participants who submitted photographs at least one year after tattooing, although the activated tattoos were less saturated than the color-matched control. Taken together, these findings indicate that a photochromic nanoparticle tattoo ink can be applied to human skin with short-term tolerability comparable to a conventional ink, and can retain reversible optical function after long-term intradermal residence.

This study has several limitations that should be considered when interpreting these findings. The cohort was small (11 participants), so the statistical power to detect infrequent adverse events or between-group differences was limited, as reflected in the Clopper–Pearson confidence interval, which permits a true adverse event rate of up to 24%. Participants were recruited by purposive sampling from the clientele of the single tattoo artist who performed all procedures, and Magic Ink was compared against a single commercial control ink, so the findings may not generalize to other populations, inks, or tattooing techniques. Safety and tolerability were assessed by dermatologist clinical inspection and participant-reported outcomes rather than by instrumented metrics such as the erythema index or transepidermal water loss, and no histological analysis of tattooed skin was performed, since the protocol was confined to minimal-risk, non-invasive assessments. The colorimetric analysis of efficacy relied on participant-submitted photographs taken under illumination and imaging conditions that were not standardized, which introduces variability that controlled imaging would reduce. Finally, the formaldehyde generated during gamma sterilization exceeded the REACH limit, so the present sterilization workflow would require further optimization for full European regulatory compliance. Future work should address these limitations through larger controlled cohorts, instrumented dermatological endpoints, histological evaluation, comparison across multiple inks and practitioners, standardized imaging, and refinement of the sterilization workflow to reduce formaldehyde generation. Within these constraints, this study provides preliminary evidence that rewritable photochromic tattoo pigments can be applied to and retained in human skin with tolerability comparable to a conventional ink and with no adverse events reported over more than three years, supporting their further evaluation for use in body art, permanent makeup, and selected medical tattooing applications.

## Experimental methods

### Materials

Magic Ink, available from https://magicink.store, was provided by HYPRSKN Inc., and used as supplied without dilution, except in the case of dilution for pigment size analysis by dynamic light scattering as detailed below. Synthetic melanin was obtained from Millipore Sigma. A SYLGARD 184 polydimethylsiloxane (PDMS) silicone resin kit was purchased from Dow Chemical. Reverse osmosis (RO)-purified water was obtained from a tap in our campus facility. All materials and solvents were used as received. Melanin-doped PDMS films of 1 mm thickness were prepared by mixing varying amounts (6–35 *μ*l) of a methanol solution of synthetic melanin (0.06 mg/*μ*l) into 1 gram of PDMS two-part (10:1) resin mixture, which was cast into plastic weigh boats and cured in a vacuum oven at 60 $^{\circ }$C overnight, then the cured films were cut to swatches of desired dimensions with a razor blade.

### Instrumentation

 For scanning electron microscopy, dilute aqueous suspensions of Magic Ink were drop-cast on a microscope slide, allowed to dry in open air, then sputter-coated with Au and imaged with a SU3500 scanning electron microscope (Hitachi). Particle size analysis by dynamic light scattering (DLS) was performed with a Nanotrac Flex particle size analyzer (Microtrac) via the backscattered laser-amplified scattering reference method using the FFT power spectrum calculation model. Prior to analysis, Magic Ink was diluted 1:200 in reverse-osmosis water and dispersed by manual shaking without sonication, filtration, or centrifugation. Since the particles are composed primarily of PMMA, the refractive index of PMMA (1.49) was used for the instrument settings. Three 30-second runs were averaged per sample, and the instrument’s Loading Index for the reported measurement was 14.8, within the 2–20 range recommended by the instrument software (v12.0.1.0) for a valid measurement. The full set of instrument parameters and raw output, including the Loading Index, fit residual, and measurement conditions, is included in Figure S1 of the Supplementary Information. UV-LED activation of Magic Ink was performed with the UV-LED lamp packaged with commercial Magic Ink for 365 nm wavelength. Longer-wavelength UV-LED lamps employed for the colorimetric wavelength dependency measurements were Mastiff B2 (375 nm), Escolite Mini UV Black Light Flashlight ESCO-0123 (385 nm), and Darkbeam V5-DB-395 nm (395 nm). Steady-shear viscosity measurements were performed with a LVDV-II+PRO CP digital viscometer (Brookfield Engineering Labs, Inc.) with medium-viscosity cone spindle CPE 51. The instrument was leveled and auto-zeroed before each measurement, and the cone-plate gap was set against the instrument’s contact-point indicator before each run. Samples were dispensed into the center of the sample cup and measured promptly to avoid drying, and were inspected to ensure the absence of air bubbles. At each rotational speed, viscosity was recorded once the reading had stabilized and the torque fell within the instrument’s reliable range of 35–65%. All measurements were repeated at least three times to verify consistent results and estimate a standard deviation of the mean.

### Colorimetry

The photochromic and colorimetric characterization reported here was performed by the authors during manuscript preparation; equivalent characterization conducted prior to the clinical study informed its design. Colorimetric readings of Magic Ink paper swatches were recorded using a PCE-XXM 30 colorimeter (PCE Instruments). For colorimetric readings of photographic data, photographs of Magic Ink pigment paper swatches were obtained on an iPhone 17 and the raw images were imported into Photoshop. Participant-submitted photographs were taken on participants’ own devices. No photographs were edited other than rotation and cropping. For colorimetric analysis, representative square-shaped samples (10x10 pixels for participant-submitted tattoo photographs, 30x30 pixels for photographs of Magic Ink paper swatches) were selected within each photograph and all pixels within the square were averaged. Then averaged L*, a*, and b* values and RGB codes of these sample areas were collected using the eyedropper tool in Adobe Photoshop software. The L*, a*, b* values are used to calculate $\Delta E_{ab}^{*}$ values from the Euclidean distance ($d=\sqrt{(L^{*}_{2}-L^{*}_{1})^{2}+(a^{*}_{2}-a^{*}_{1})^{2}+(b^{*}_{2}-b^{*}_{1})^{2}}$) between two points in CIELAB chromaticity space. In participant-submitted photographs, the above sampling procedure was accompanied by a whole-tattoo averaging procedure to calculate $(\Delta E_{ab}^{*})_{avg}$ values. The edges of the tattoos were selected manually in Photoshop and all pixels within the whole heart-shaped tattoo area and were averaged and compared with the average obtained from the entire non-tattooed area inside the heart shape; $(\Delta E_{ab}^{*})_{avg}$ values were then obtained from the Euclidean distance between these monochromatic whole-tattoo-averaged L*-a*-b* values. $(\Delta E_{ab}^{*})_{max}$ was calculated analogously by averaging five 10x10 pixel squares selected from the darkest parts of the tattoo and five equidistant samples of bare skin within the tattoo outline.

### Spectrometry and dosage estimations

UV/Vis absorbance spectra were recorded of a highly dilute Magic Ink suspension in reverse-osmosis (RO) water in a quartz cuvette on a CARY UV-Vis 60 spectrophotometer. Spectral power distributions were recorded using a USB digital spectrometer (Aimed Research) and Theremino Spectrometer v2.5 software. For LED sources, the integrated radiant flux of UV light was estimated using a calibrated UVA/B radiometer (UVA/B Light Meter 850009, Sper Scientific), with a UV detection range of 290–380 nm and a peak sensitivity at 350 nm wavelength. The wavelength-dependent irradiance of the UV-LED source was then obtained by fitting the observed spectral power distribution to a magnitude that integrates to the total flux observed radiometrically. For white light sources, luminous flux was measured in lux with a digital light meter (LOMEDRETME model S8608). To represent average sunlight for comparison of radiant power and dosage, we employed the ASTM G173-03 standard solar power spectrum [100] and the CIE McKinlay-Diffey action spectrum. [101] The erythemal-weighted action spectra of both UV-LED and solar sources were calculated in Microsoft Excel and the integrals were approximated therein using Riemann sums of 1-nm resolution.

### Fatigue resistance

In the fatigue resistance experiments, colorimeter readings were recorded in triplicate for three sampled areas of the swatch and averaged (total 9 readings) after each 1000-cycle on/off exposure period, which was automated with a custom-built UV/Vis irradiation cycling tool that automatically switches between 15 s UV-LED irradiation and 180 s white LED irradiation controlled by a Raspberry Pi.

### Statistical analysis

All statistical analysis was performed using Microsoft Excel Software. Paired binary survey responses for pain, redness, and itch (each analyzed separately as present vs absent) were compared between Magic Ink and Standard Ink tattoos using exact McNemar tests at each timepoint, with two-sided p-values computed from discordant pairs under a binomial model. Colorimetric experiments were performed at least three times, and the data are expressed as the mean ± standard deviation (SD) for the total number of experiments. Differences in color between Magic Ink tattoos and adjacent skin before and after UV-LED exposure, quantified by $\Delta E_{ab}^{*}$, were analyzed using a paired Student’s t-test. p values < 0.05 were considered statistically significant.

## Additional file

Supplementary file 1

## Data Availability

The datasets supporting the conclusions of this article are included within the article and the Supplementary Information file.
