# Supplementary material for: Clinical evaluation of photochromic nanoparticle tattoo ink: safety, tolerability, and performance of rewritable intradermal implants
Source: J Nanobiotechnology. 2026 Jul 2;24:894. doi: 10.1186/s12951-026-04755-x (PMC13599091; doi:10.1186/s12951-026-04755-x)
Supplement: Supplementary file 1 [file 12951_2026_4755_MOESM1_ESM.pdf]

Supplementary Information

Clinical Evaluation of Photochromic Nanoparticle Tattoo Ink: Safety, Tolerability, and Performance of Rewritable Intradermal Implants

Keith McCurdy[1], Jesse L. Butterfield[1], Hyejin Kwon[1,2], Emily Gardner[1,2], Madison Jordan[1], Matthew Ganser[1], Michael J Mouncer[1], Jon Osis[1], Christophe Clement[1], Adarsh V. Mudgil[*1,4], and Carson J. Bruns[*1,2,3]

[1]HYPRSKN, Inc.
[2]ATLAS Institute, University of Colorado Boulder
[3]Rady Department of Mechanical Engineering, University of Colorado Boulder
[4]Mudgil Dermatology, New York City
[*]carson.bruns@colorado.edu; carson.bruns@hyprskn.com
[*]amudgil@mudgildermatology.com

a. Number-Weighted Distribution

MICROTRAC MRB

Particle Size Analysis

3/24/2023 11:09 AM

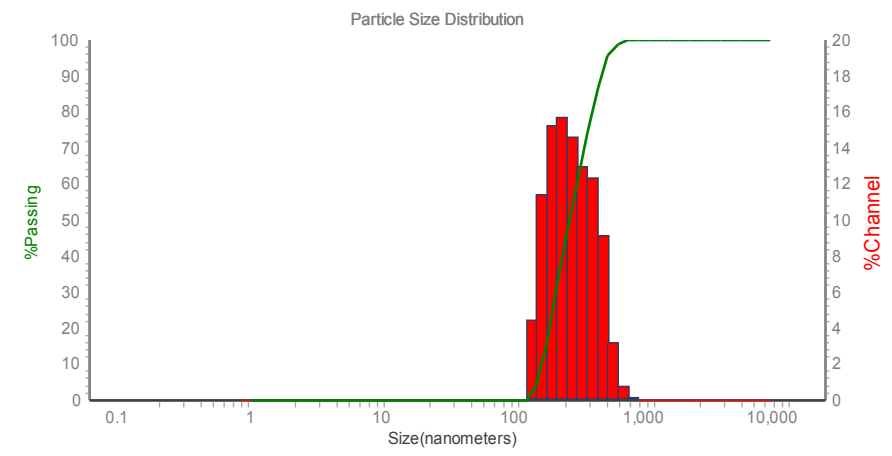

Summary	
Data	Value
MV(nm):	386.0
MN(nm):	234.6
MA(nm):	315.0
CS:	19.05
SD:	92.80
PDI:	0.0991
Mz:	344.9
si:	0.1213
Ski:	0.1070
Kg:	1.254

Percentiles	
%Tile	Size(nm)
10.00	133.6
20.00	151.8
30.00	169.8
40.00	189.4
50.00	212.0
60.00	238.9
70.00	272.5
80.00	312.0
90.00	363.0
95.00	401.0

Peaks Summary		
Dia(nm)	Vol%	Width
212	100	185.6

Tabular Data

Size(nm)	%Chan	%Pass	Size(nm)	%Chan	%Pass
6540	0.00	100.00	12.77	0.00	0.00
5500	0.00	100.00	10.74	0.00	0.00
4620	0.00	100.00	9.03	0.00	0.00
3890	0.00	100.00	7.60	0.00	0.00
3270	0.00	100.00	6.39	0.00	0.00
2750	0.00	100.00	5.37	0.00	0.00
2312	0.00	100.00	4.52	0.00	0.00
1944	0.01	100.00	3.80	0.00	0.00
1635	0.01	99.99	3.19	0.00	0.00
1375	0.00	99.98	2.690	0.00	0.00
1156	0.00	99.98	2.260	0.00	0.00
972.0	0.01	99.98	1.900	0.00	0.00
818.0	0.03	99.97	1.600	0.00	0.00
687.0	0.13	99.94	1.340	0.00	0.00
578.0	0.79	99.81	1.130	0.00	0.00
486.0	3.24	99.02	0.950	0.00	0.00
409.0	9.13	95.78			
344.0	12.32	86.65			
289.0	12.95	74.33			
243.0	14.58	61.38			
204.4	15.67	46.80			
171.9	15.25	31.13			
144.5	11.43	15.88			
121.5	4.45	4.45			
102.2	0.00	0.00			
85.90	0.00	0.00			
72.30	0.00	0.00			
60.80	0.00	0.00			
51.10	0.00	0.00			
43.00	0.00	0.00			
36.10	0.00	0.00			
30.40	0.00	0.00			
25.55	0.00	0.00			
21.48	0.00	0.00			
18.06	0.00	0.00			
15.19	0.00	0.00			

WARNING: NONE

Measurement Info

Title	
Particle Size Analysis	
Identifiers	
10ofHearts_Ster	
EG	
Database Record	58
Run Number	Avg of 3
Date	3/24/2023
Time	10:13 AM
Acquired Date	3/23/2023
Acquired Time	3:52 PM
Serial Number	W3803
Calc SW Ver	12.0.1.0
Calculated Data	
Residuals, Above:Below	0:0
Loading Index	1.48E+1
Conc. Index	0.882
Concentration cc/ml	5.15E-4
RMS Residual	0.125%
Cell Temp (C)	21.97
Viscosity(cp)	0.9560
Reflected Pwr (uW)	1.70
User Defined Calculations	
Name	Value
Recalculation Status	
DB-Meas : : :	

SOP Info

EG1(*)	
Timing	
Setzero Time	30 (sec)
Run Time	90 (sec)
Number of Runs	3
Multi-Run Delay	0 (min)
Delay First Meas.	Disabled
Analysis	
pmma	
Refractive Index	1.49
Transparency	Transp
Shape	Spherical
WATER	
Refractive Index	1.33
Low Temperature	20.0
Low Temp. Visc.	1.002
High Temperature	30.0
High Temp. Visc.	0.797
Options:	
Analysis Type	Distribution
Filter:Resolution	Std:Norm
Sensitivity	Standard
Algorithm:	2.0
Perspective	
Progression	Standard
Distribution	Number
Upper Edge(nm)	6540
Lower Edge(nm)	0.8
Residuals	Disabled

b. Volume-Weighted Distribution

MICROTRAC MRB

Particle Size Analysis

3/24/2023 11:07 AM

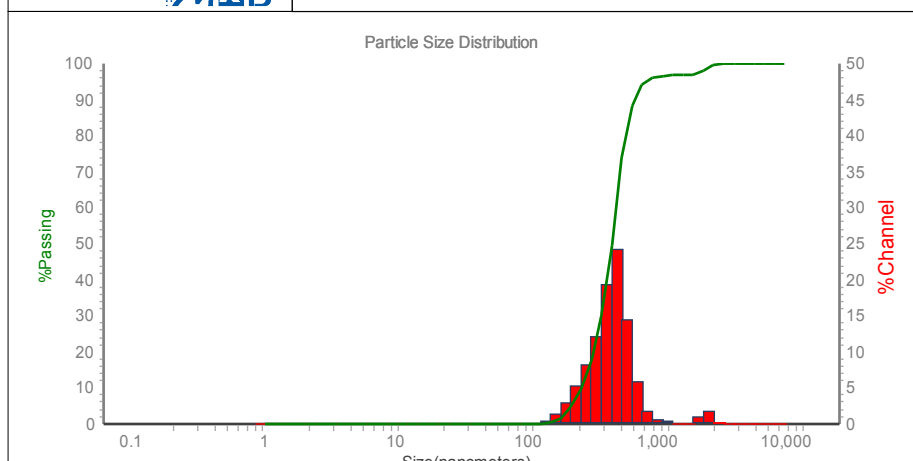

Summary	
Data	Value
MV(nm):	386.0
MN(nm):	234.6
MA(nm):	315.0
CS:	19.05
SD:	110.6
PDI:	0.0991
Mz:	344.9
si:	0.1213
Ski:	0.1070
Kg:	1.254

Percentiles	
%Tile	Size(nm)
10.00	205.1
20.00	251.5
30.00	288.5
40.00	318.0
50.00	345.0
60.00	370.0
70.00	397.0
80.00	434.0
90.00	504.0
95.00	610.0

Peaks Summary		
Dia(nm)	Vol%	Width
1722	3.1	365
341	96.9	209.2

Tabular Data

Size(nm)	%Chan	%Pass	Size(nm)	%Chan	%Pass
6540	0.00	100.00	12.77	0.00	0.00
5500	0.00	100.00	10.74	0.00	0.00
4620	0.00	100.00	9.03	0.00	0.00
3890	0.00	100.00	7.60	0.00	0.00
3270	0.00	100.00	6.39	0.00	0.00
2750	0.00	100.00	5.37	0.00	0.00
2312	0.27	100.00	4.52	0.00	0.00
1944	1.84	99.73	3.80	0.00	0.00
1635	0.93	97.89	3.19	0.00	0.00
1375	0.05	96.96	2.690	0.00	0.00
1156	0.00	96.91	2.260	0.00	0.00
972.0	0.35	96.91	1.900	0.00	0.00
818.0	0.63	96.56	1.600	0.00	0.00
687.0	1.70	95.93	1.340	0.00	0.00
578.0	5.92	94.23	1.130	0.00	0.00
486.0	14.48	88.31	0.950	0.00	0.00
409.0	24.27	73.83			
344.0	19.41	49.56			
289.0	12.15	30.15			
243.0	8.14	18.00			
204.4	5.20	9.86			
171.9	3.01	4.66			
144.5	1.34	1.65			
121.5	0.31	0.31			
102.2	0.00	0.00			
85.90	0.00	0.00			
72.30	0.00	0.00			
60.80	0.00	0.00			
51.10	0.00	0.00			
43.00	0.00	0.00			
36.10	0.00	0.00			
30.40	0.00	0.00			
25.55	0.00	0.00			
21.48	0.00	0.00			
18.06	0.00	0.00			
15.19	0.00	0.00			

WARNING: NONE

Measurement Info

Title	
Particle Size Analysis	
Identifiers	
10ofHearts_Ster	
EG	
Database Record	8
Run Number	Avg of 3
Date	3/23/2023
Time	3:52 PM
Acquired Date	3/23/2023
Acquired Time	3:52 PM
Serial Number	W3803
Calc SW Ver	12.0.1.0
Calculated Data	
Residuals, Above:Below	0:0
Loading Index	1.48E+1
Conc. Index	0.882
Concentration cc/ml	5.15E-4
RMS Residual	0.125%
Cell Temp (C)	21.97
Viscosity(cp)	0.9560
Reflected Pwr (uW)	1.70
User Defined Calculations	
Name	Value
Recalculation Status	
DB-Meas : : Original :	

SOP Info

EG1(*)	
Timing	
Setzero Time	30 (sec)
Run Time	90 (sec)
Number of Runs	3
Multi-Run Delay	0 (min)
Delay First Meas.	Disabled
Analysis	
pmma	
Refractive Index	1.49
Transparency	Transp
Shape	Spherical
WATER	
Refractive Index	1.33
Low Temperature	20.0
Low Temp. Visc.	1.002
High Temperature	30.0
High Temp. Visc.	0.797
Options:	
Analysis Type	Distribution
Filter:Resolution	Std:Norm
Sensitivity	Standard
Algorithm:	2.0
Perspective	
Progression	Standard
Distribution	Volume
Upper Edge(nm)	6540
Lower Edge(nm)	0.8
Residuals	Disabled

Figure S1: Raw data output files from particle size analysis by dynamic light scattering (DLS).

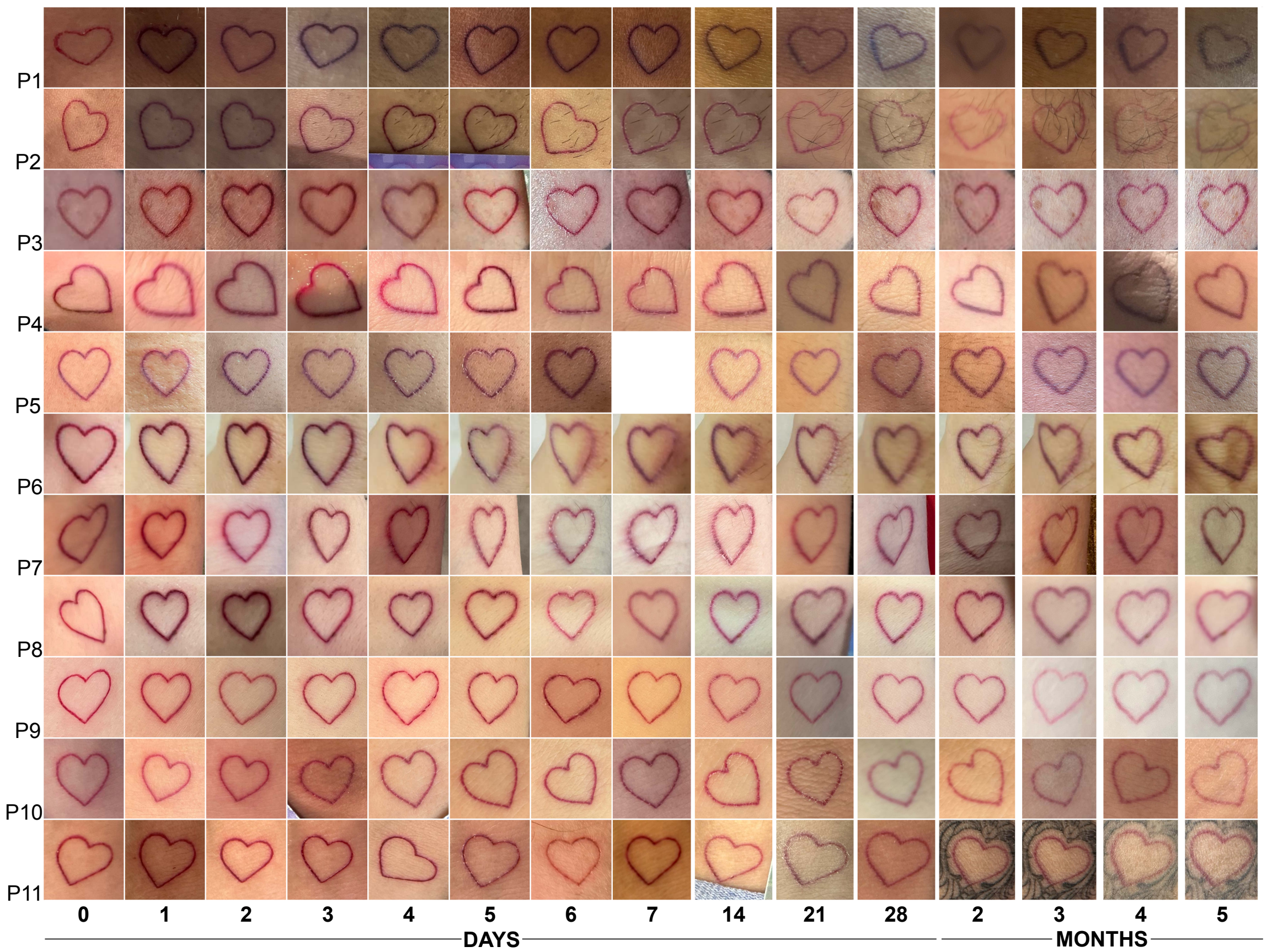

Figure S2: Timeline of participant-submitted photos of Standard Ink tattoos.

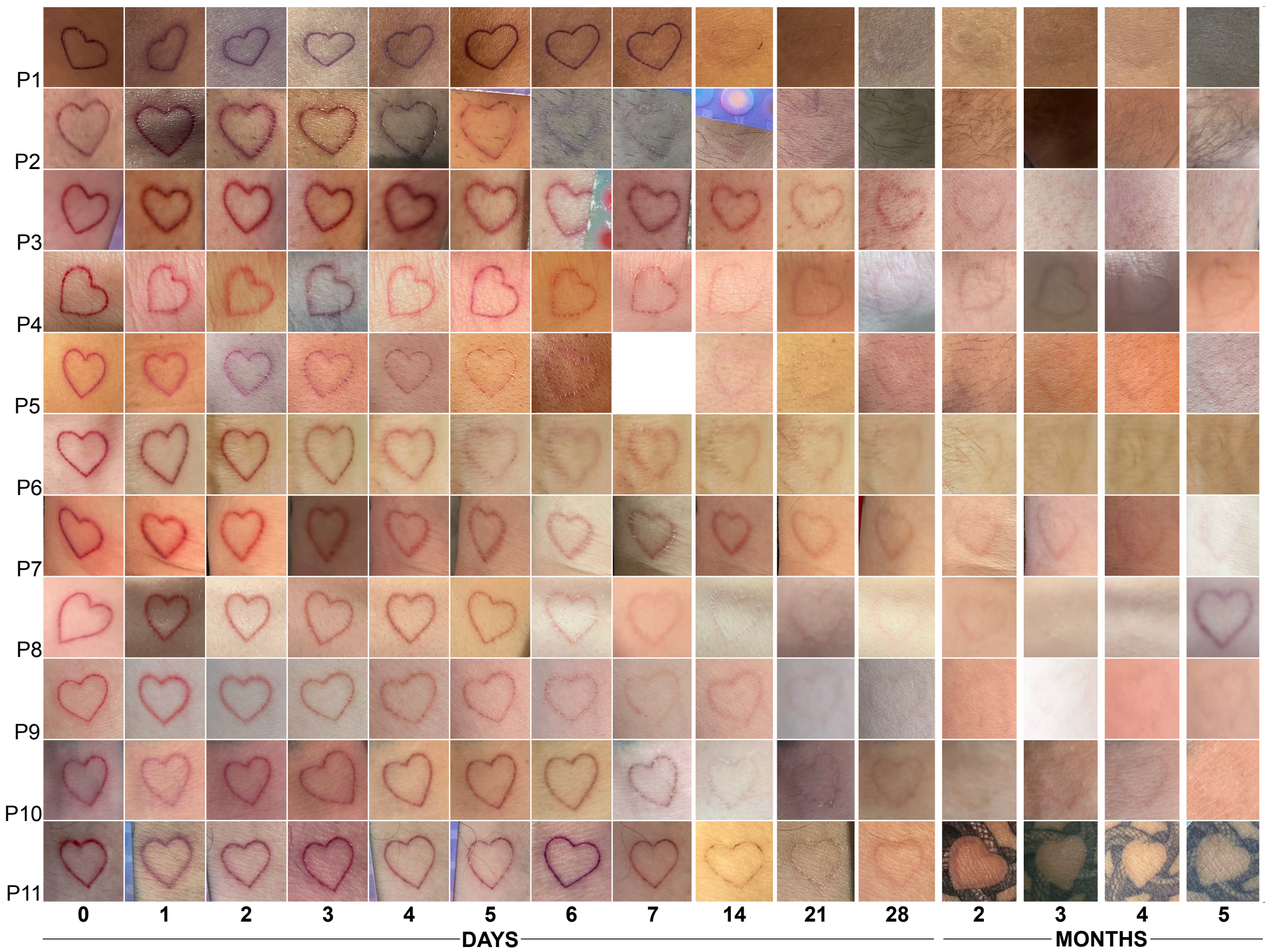

Figure S3: Timeline of participant-submitted photos of Magic Ink tattoos.

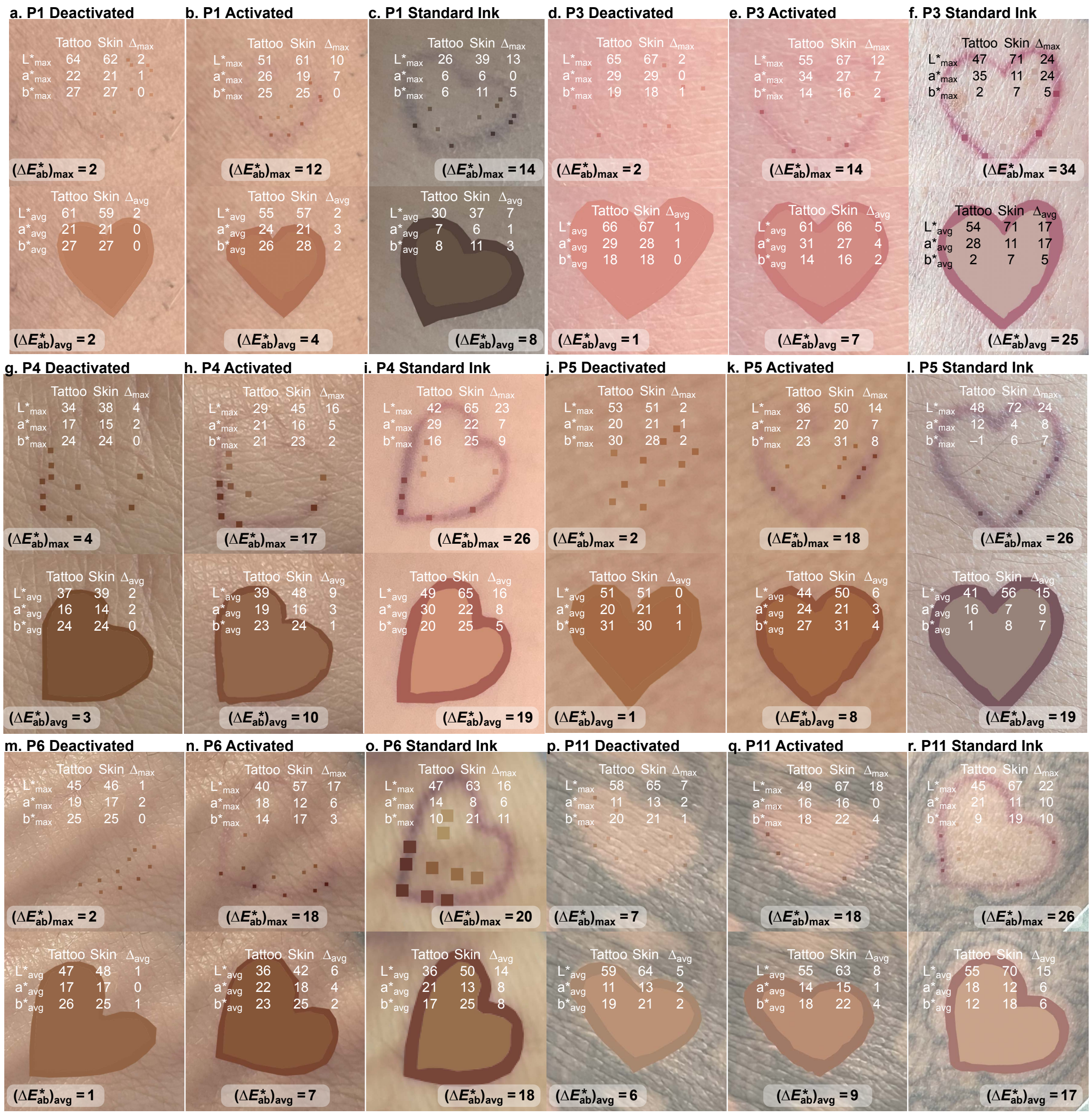

Figure S4: Colorimetric data for calculating $(\Delta E^*_{ab})_{max}$ and $(\Delta E^*_{ab})_{avg}$ for deactivated and UV-activated Magic Ink tattoos at 12 months and Standard Ink control tattoos at 5 months.

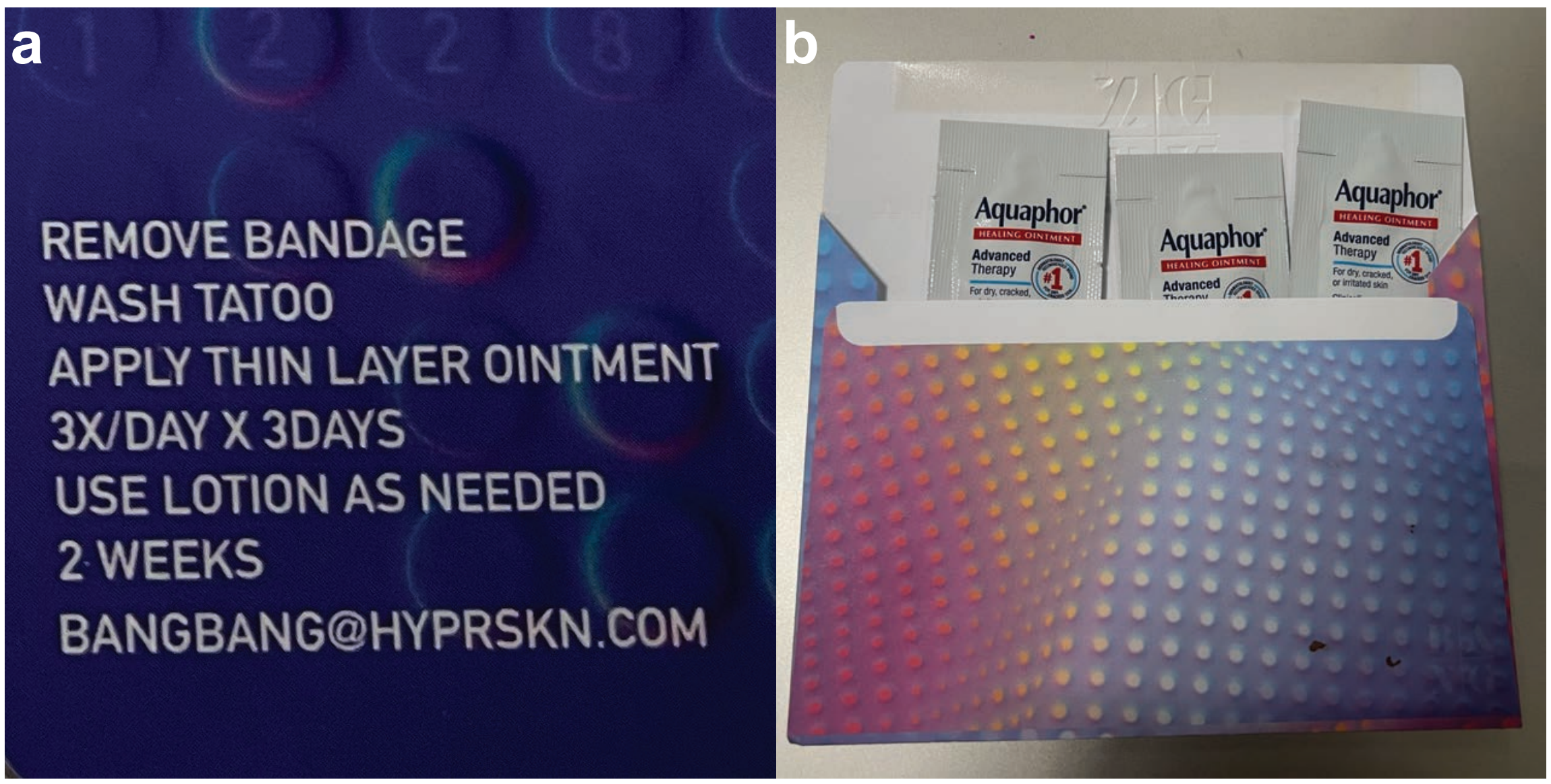

Figure S5: Photographs of (a) aftercare instructions and (b) ointment provided to all participants. Participants were instructed to apply a thin layer of Aquaphor three times a day for the first three days, then to apply lotion (not provided) as needed for two weeks. Aquaphor is a standard occlusive emollient used to keep the healing tattoo moist and protected during the initial inflammatory phase.
